# Immunotherapy-Based Therapeutic Strategies for Recurrent Advanced Squamous Cell Carcinoma of the Head and Neck: A Case Report and Literature Review

**DOI:** 10.3389/fimmu.2021.680327

**Published:** 2021-07-21

**Authors:** Hao Nie, Ting Chen, Kefei He, Chanjin Liang, Wei Guo, Xingyuan Shi

**Affiliations:** Department of Radiation Oncology, The Fifth Affiliated Hospital of Guangzhou Medical University, Guangzhou, China

**Keywords:** case report, locoregionally advanced squamous cell carcinoma of head and neck, targeted therapy, immunotherapy, radiotherapy

## Abstract

We present a patient with locoregionally advanced laryngeal carcinoma, who experienced recurrence 2 months after surgery. We exploratively treated this patient with immunotherapy combined with targeted therapy with or without radiation therapy. The patient exhibited a significant and durable response. Thus far, there are no standard or effective second-line therapeutic modalities for recurrent locoregionally advanced laryngeal carcinoma. The efficacy of conventional chemotherapy with anti-epidermal growth factor receptor (anti-EGFR) remains unsatisfactory. The addition of immunotherapy resulted in substantial improvement in the progression-free survival (PFS) and overall survival (OS) of this patient. In this case, immunotherapy combined with anti-EFGR was administered, leading to good tumor response; based on this observation, radiotherapy was added to further intensify tumor control. This therapeutic strategy may be a novel option for recurrent locoregionally advanced squamous cell carcinoma of the head and neck.

## Introduction

Over 800,000 new cases of head and neck squamous cell carcinoma (HNSCC) occur annually worldwide, and the mortality rate associated with this disease is approximately 40–50% (1). Particularly for patients with recurrent or metastatic (R/M) HNSCC, the expected survival periods tend to be <1 year due to limited treatment options. Most patients with R/M HNSCC undergo palliative systematic treatment and best supportive care, with only a minority having the chance to receive radical local treatment (e.g., surgery and radiotherapy). According to the EXTREME protocol and Chinese CHANGE-2 study, the recommended first-line therapy involves platinum and 5-fluorouracil (5-FU)-based chemotherapy combined with cetuximab. It has been shown that this regimen significantly enhances the tumor regression rate, reduces the progression rate, and extends overall survival, thereby, improving the quality of life of patients. In recent years, an immunotherapy based on checkpoint inhibitors has been linked to important developments in the treatment of advanced HNSCC. The U.S. Food and Drug Administration approved the use of nivolumab and pembrolizumab for the salvage treatment of R/M HNSCC. Based on the results of the KEYNOTE-048 study, the National Comprehensive Cancer Network has listed immunotherapy (as monotherapy or in a combination regimen) as one of the first-line treatment options for R/M HNSCC. However, we observed that, at the initial stage, the objective response rate (ORR) and progression-free survival of patients undergoing immunotherapy are similar to those reported in the EXTREME strategy. Herein, we present a case of R/M HNSCC who was treated with the exploratory combination of immunotherapy and targeted therapy, followed by radiotherapy, achieving encouraging results. Furthermore, we also reviewed the relevant literature.

## Case Presentation

**Figure 1:**
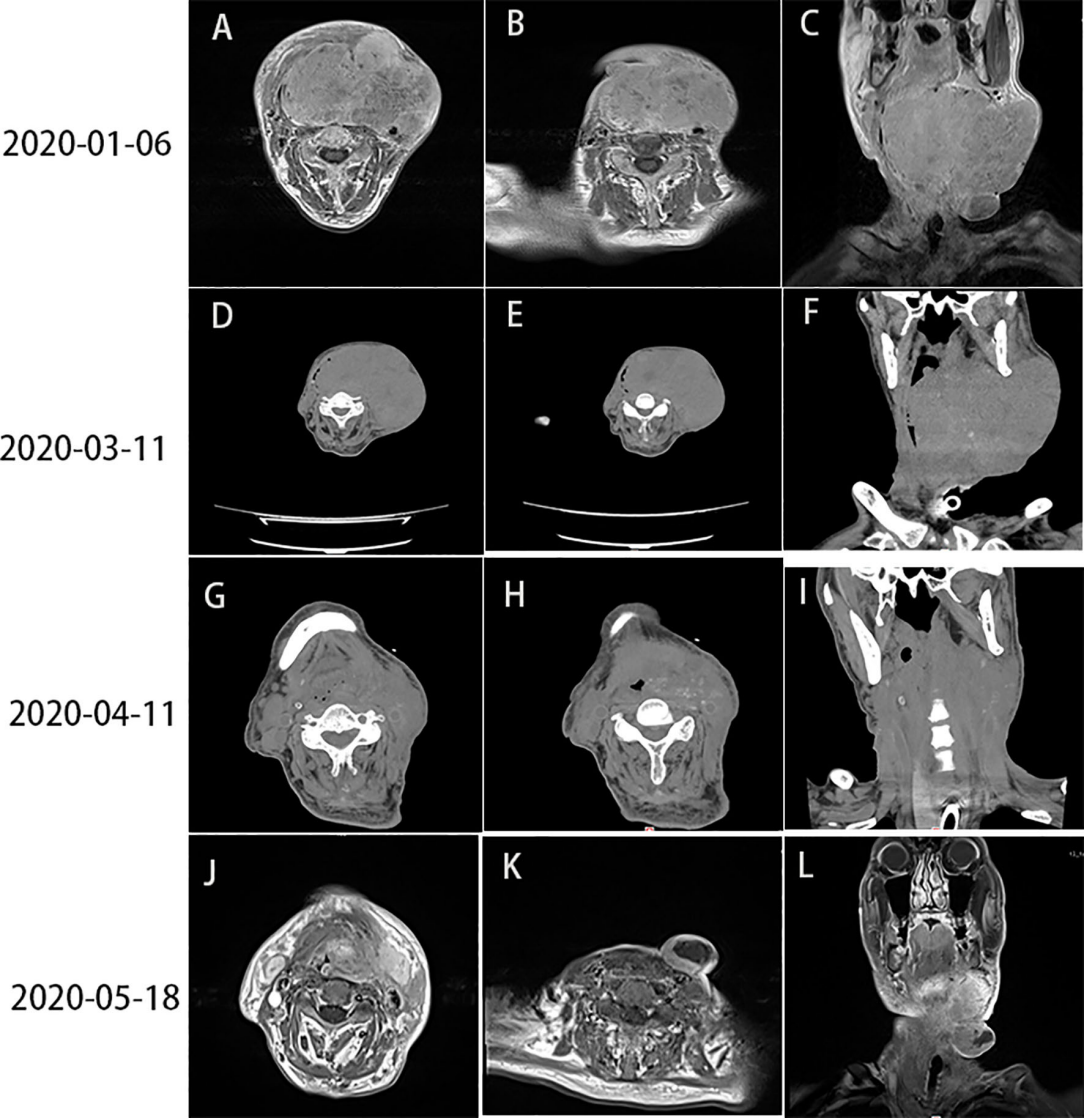
**(A–C)** Head and neck CT showed lymph node enlargement (10 × 12 cm) on the left side of the neck (January 6, 2020). **(D–F)** Head and neck CT, performed 10 days after the first cycle of treatment, showed progression of the tumor mass (13 × 10 cm) (March 11, 2020). **(G–I)** PR was detected after the first course of combination therapy with sintilimab and nimotuzumab (April 11, 2020). **(J–L)** Head and neck MRI revealed PR (May 18, 2020). CT, computed tomography; MRI, magnetic resonance imaging; PR, partial response.

In July, 2019, a 64-year-old male was admitted to the Guangdong Provincial People’s Hospital complaining of a lump found on the left side of his neck. Neck magnetic resonance imaging (MRI) revealed an irregular mass in the left glottic region involving the vocal cords, posterior commissure, left epiglottis, and thyroid cartilage, which indicated glottic carcinoma with left neck lymph node infiltration. A pathological analysis through a laryngoscopic biopsy indicated squamous carcinoma of the larynx. The definitive diagnosis was larynx squamous cell carcinoma cT4N3M0, stage IV. On July 17 and August 9, 2019, the patient underwent two cycles of induction chemotherapy with docetaxel and cisplatin combined with 5-FU, which resulted in a stable disease. Subsequently, the patient underwent a radical laryngectomy and a cervical lymph node dissection in September, 2019, but he refused to receive adjuvant chemoradiotherapy. In November, 2019, the patient was admitted to our hospital with enlarged left neck lymph nodes. A head and neck computed tomography (CT) following admission revealed a left neck lymph node enlargement (10 × 12 cm) (**Figures 1A–C**). A thorax and abdomen CT (on January 6, 2020) did not show lung or liver metastasis. A biochemical analysis of blood revealed increased levels of creatinine (228 umol/L), sodium (156 mmol/L), and calcium (4.02 mmol/L). The patient was diagnosed with larynx squamous carcinoma cT4N3M0, stage IV (according to the eighth edition of the American Joint Committee on Cancer TNM staging); this was recurrent after surgery and accompanied by renal insufficiency and hypercalcemia.

The patient had difficulty in food intake due to tumor compression. On January 8, 2020, gastrostomy was performed to relieve this symptom and improve the patient’s state of nutrition. Treatment selection was based on the National Comprehensive Cancer Network guidelines, the results of the EXTREME and CHANGE-2 studies, and the poor general condition of the patient. Owing to its good safety profile, albumin paclitaxel in combination with nimotuzumab 200 mg was the selected regimen. The patient underwent two cycles of treatment between January 15 and February 17, 2020. A testing of electrolytes in blood suggested intractable hypernatremia and hypercalcemia. Hence, the patient underwent continuous renal replacement therapy. A reexamination after two courses of the treatment revealed significant enlargement of the neck mass, which indicated progressive disease.

**Figure 2:**
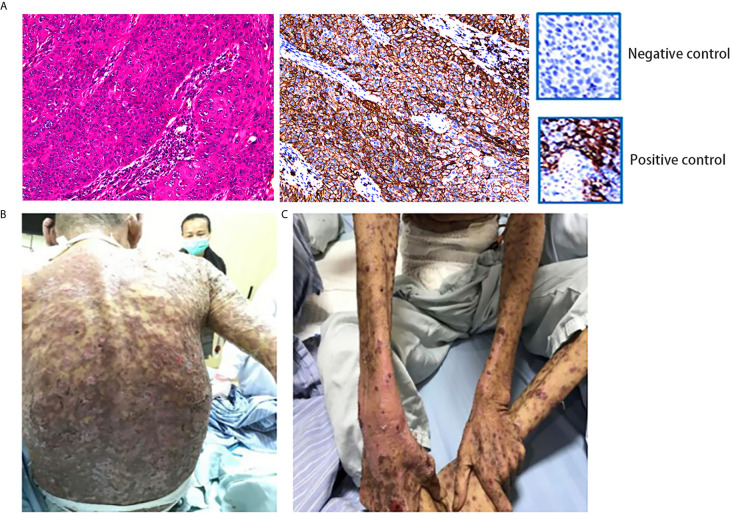
**(A)** IHC showed positivity for PD-L1. (Dako 22C3 antibody was used to provide positive control and negative control.) This specimen was sliced from formalin-fixed paraffin-embedded tissue. **(B, C)** The patient developed grade IV drug-induced dermatitis after the first course of combination therapy with sintilimab and nimotuzumab. HE, hematoxylin–eosin; IHC, immunohistochemistry; PD-L1, programmed death-ligand 1.

To further seek other treatment options, the patient consented to undergo next-generation sequencing, which covered 428 genes and an immunohistochemical analysis of programmed death-ligand 1 (PD-L1). The outcome of next-generation sequencing showed a low tumor mutational burden (11.5 mut/Mb), and mutations in genes F-box and WD repeat domain containing 7 (*FBXW7*), *PKHD1*, and FAT atypical cadherin 1 (*FAT1*). The results of the immunohistochemical analysis yielded a combined positive score (CPS) of 95 and a tumor proportion score of 95% for PD-L1, which indicated positivity (**Figure 2A**).

Based on the results of two major clinical trials, namely KEYNOTE-048 and KEYNOTE-040, an immunotherapy was considered for this patient. On February 29, 2020, the patient received the first cycle of treatment with albumin paclitaxel (100 mg) in combination with sintilimab (200 mg). However, a reexamination using a head and neck CT on March 11, 2020 revealed progression of the tumor mass (13 × 10 cm) (**Figures 1D–F**).

Despite previous treatment, the tumor remained uncontrolled. Moreover, the general condition of the patient was deteriorating. We reviewed clinical trials involving combinations of immunotherapy and targeted therapy using the PubMed database (National Institutes of Health, Bethesda, MD, USA). Most of them were phase II studies without declared preliminary results. We communicated with the family of the patient and suggested using the combination of immunotherapy and targeted therapy. On March 20, 2020, the patient received sintilimab (200 mg, once every 3 weeks) and nimotuzumab (200 mg, once weekly). Four days after the treatment, the patient developed grade IV drug dermatitis (**Figures 2B, C**). The patient recovered after treatment with glucocorticoids, antiallergic agents, and other symptomatic treatments (e.g., relief of itching and promotion of mucosal repair).

On April 11, 2020, a head and neck CT revealed regression of the tumor mass (6 × 7 cm) and partial response (PR) to the therapy (**Figures 1G–I**).

The general condition of the patient improved 1 month later. Subsequently, he received two additional cycles of sintilimab (200 mg, once every 3 weeks) and nimotuzumab (100 mg once weekly) from April 24 to May 18, 2020. Considering that the dermatitis had not been completely resolved, the dose of nimotuzumab was reduced by half. The patient was reexamined on May 18, 2020; head and neck MRI showed a maximum tumor mass diameter of 3 × 4 cm and PR to therapy (**Figures 1J–L**).

**Figure 3:**
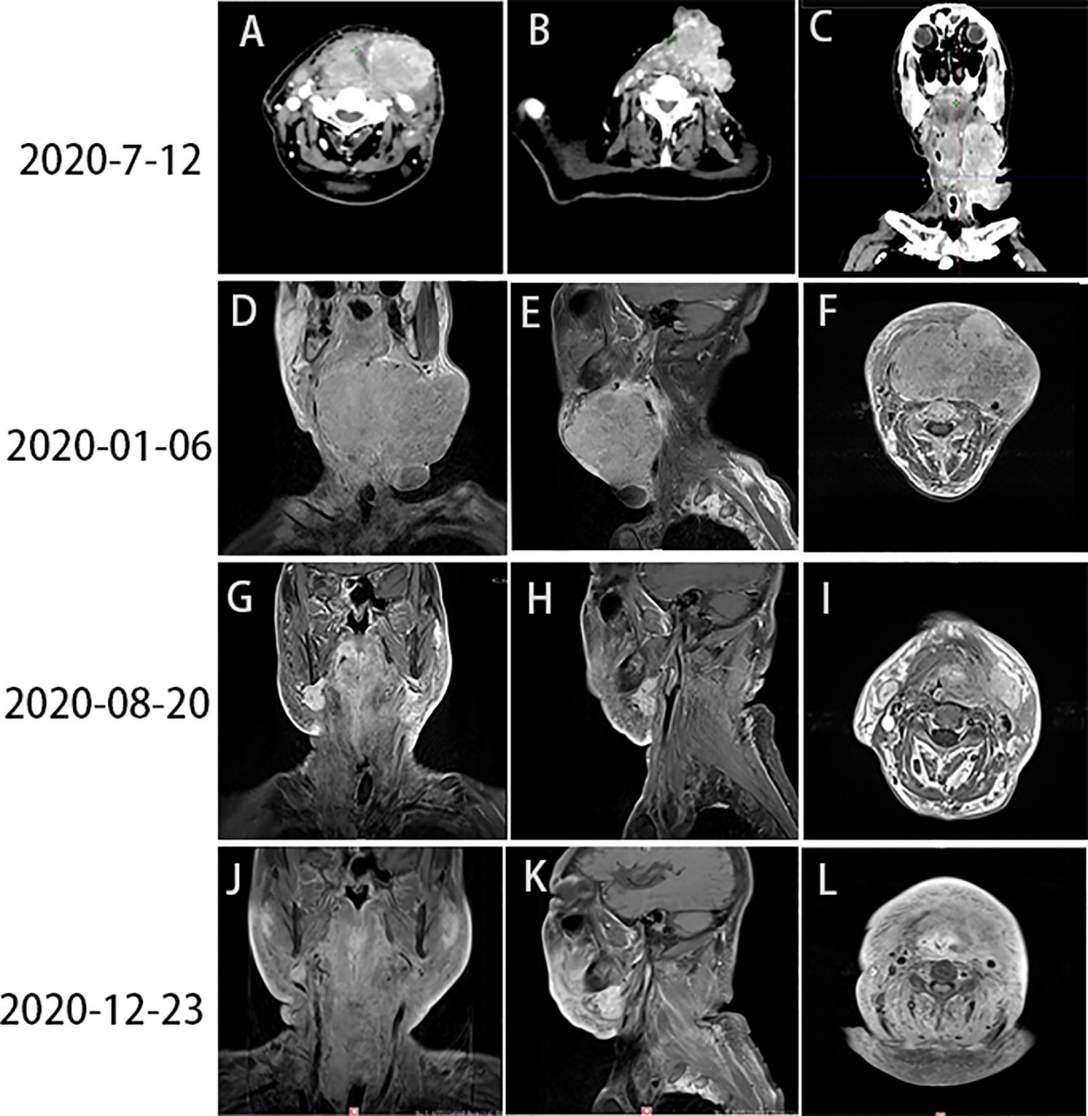
**(A–C)** Head and neck CT revealed PD (June 12, 2020). **(D–F)** Images captured prior to radiotherapy (January 6, 2020). **(G–I)** Images captured after the end of radiotherapy (August 20, 2020). **(J–L)** The tumor mass had almost completely regressed by December 23, 2020. CT, computed tomography; PD, progressive disease.

On June 12, 2020, the patient underwent a routine review. Unfortunately, physical examination revealed an enlargement of the tumor mass (**Figures 3A–C**).

Subsequently, radiotherapy of the cervical lesions was performed. The intensity-modulated radiation therapy plans were adopted (dose total: planning gross tumor volume: 64 Gy/31 F; planning clinical tumor volume: 56 Gy/31 F). On June 16, 2020, the patient underwent concurrent treatment with sintilimab (200 mg, once every 4 weeks) and nimotuzumab (200 mg, once weekly). From July 7 to August 14, 2020, he received two cycles of treatment with sintilimab (200 mg, once every 4 weeks) and nimotuzumab (200 mg, once every 2 weeks). On August 13, 2020, a boost radiotherapy dose (dose total: planning gross tumor volume: 10 Gy/4 F) was administered for residual lesions. The patient completed the radiotherapy on August 20, 2020. Compared to baseline imaging data before radiotherapy (**Figures 3D–F**), reexamination revealed that the size of the neck mass was significantly reduced (**Figures 3G–I**).

**Figure 4:**
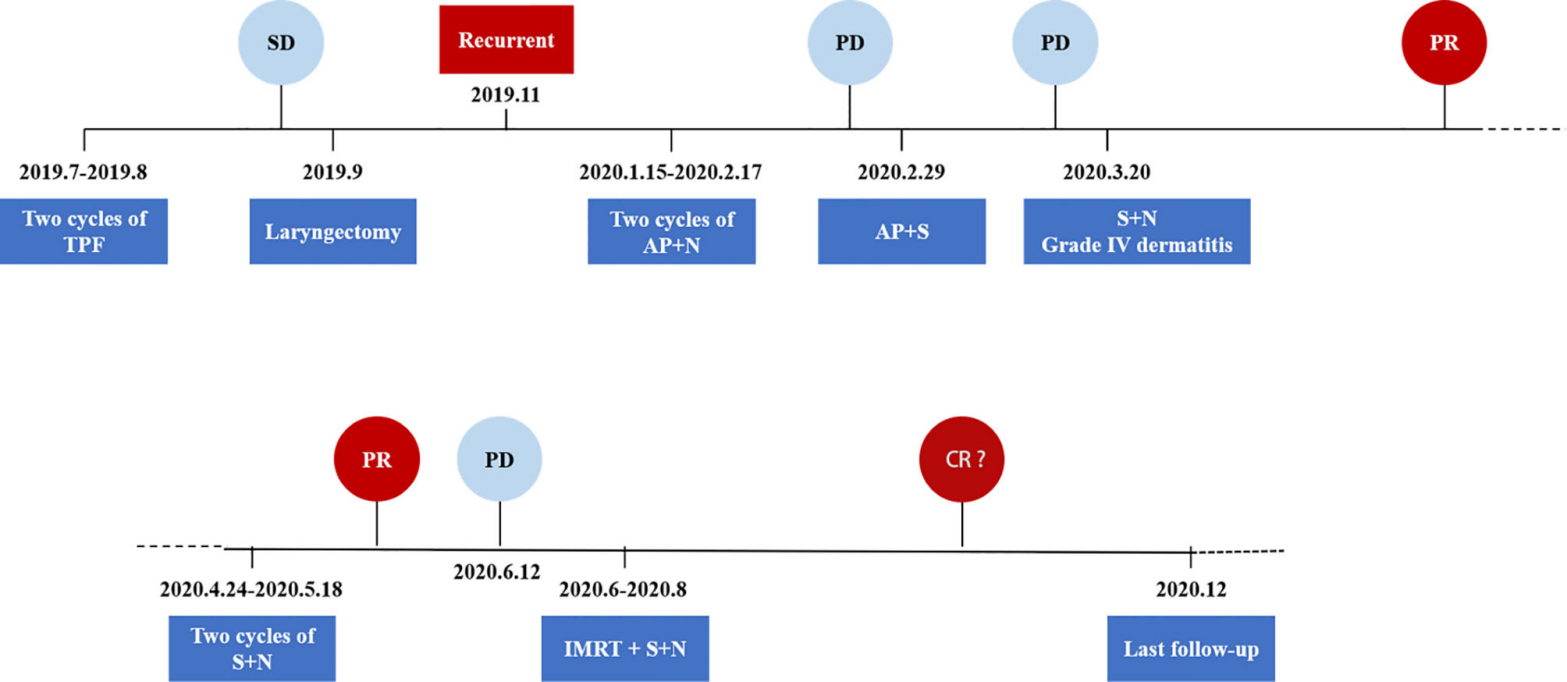
Schematics and timeline of treatment. AP, albumin paclitaxel; CR, complete response; N, nimotuzumab; PD, progressive disease; PR, partial response; S, sintilimab; SD, stable disease; TPF, docetaxel, cisplatin, and 5-fluorouracil.

The last follow-up visit of this patient was conducted on December, 2020. His general state was markedly improved, and the Karnofsky Performance Status score was 80. A head and neck MRI revealed that the neck mass had nearly disappeared (**Figures 3J–L**). The entire treatment process and disease status of the patient are summarized in **Figure 4**.

## Discussion

### Overall Prognosis of R/M HNSCC

Head and neck carcinoma is the seventh most common type of cancer worldwide, with approximately 800,000 new cases and >500,000 deaths reported annually (1). More than 65% of patients with this disease may develop R/M HNSCC, which was considered incurable in the past (2).

### Interpretation of the Classic EXTREME and Chinese CHANGE-2 Studies *Versus* the Outcome of the Present Case

Before the advent of immunotherapy, the EXTREME trial was the first to investigate the addition of targeted therapy to the traditional chemotherapy regimen. This study established the combination of cetuximab (EGFR monoclonal antibody) with 5-FU and platinum for the first-line treatment of R/M HNSCC. Following the addition of the EGFR monoclonal antibody, the OS of patients was extended from 7.4 to 10.1 months,; the PFS was also extended by 2.3 months compared with chemotherapy alone (5.6 *vs.* 3.3 months, respectively). The CHANGE-2 study, involving a Chinese population, lowered the drug dose on the basis of the EXTREME study. The results of both studies were similar. The PFS and OS of patients in the CHANGE-2 study were significantly prolonged (5.5 months *vs.* 4.2 months and 10.2 months *vs.* 8.4 months, respectively). The ORR of patients who underwent chemotherapy combined with cetuximab was 50%; this rate was significantly higher than that of patients who underwent chemotherapy alone (26.6%). With reference to the results of the international EXTREME study and the Chinese CHANGE-2 study, the patient in this case was treated with nimotuzumab (EGFR monoclonal antibody) combined with chemotherapy (albumin paclitaxel). However, the effect was not significant, and progressive disease was detected after approximately 1 month.

### Interpretation of Advances in Immunotherapy for HNSCC *Versus* the Present Case

In the 10 years following the approval of targeted therapy, the emergence of immunotherapy has further improved the survival and prognosis of patients with R/M HNSCC. The early KEYNOTE-012 phase Ib study and CheckMate-141 phase III clinical trial showed that single-agent immunotherapy as second-line treatment significantly prolonged the OS of patients with R/M HNSCC (3, 4), thereby supporting the use of immunotherapy in this setting. The KEYNOTE-048 study included 882 untreated R/M HNSCC patients with positive PD-L1 expression (CPS ≥1/CPS ≥20). The study compared single-agent immunotherapy, immunotherapy combined with chemotherapy, and the classic regimens used in the EXTREME study. The results showed that single-agent immunotherapy with pembrolizumab was associated with fewer adverse reactions compared with the EXTREME study regimens. Moreover, both single-agent immunotherapy and the combination therapy of chemotherapy and immunotherapy showed a longer OS *versus* the EXTREME study regimen. Among the study population, the patients with a CPS ≥20 exhibited a higher 4-year OS than those with a CPS ≥1 (28.6% *vs.* 19.4%, respectively), suggesting that higher expression of PD-L1 may be associated with longer OS. The present patient had a CPS of 95, which indicated high expression of PD-L1; hence, the patient belonged to the population that can benefit from immunotherapy. Therefore, treatment with sintilimab (200 mg) combined with albumin paclitaxel (100 mg) was initiated. However, the tumor was enlarged after 2 weeks, possibly due to insufficient activation of the immune system. The present case demonstrates that patients with high expression of immune biomarkers may have better disease control following treatment with the combination of chemotherapy and immunotherapy.

### Interpretation of the Progress in the Feasibility and Safety of Clinical Trials of Immunotherapy Combined With Targeted Therapy for Advanced HNSCC

Regimens without chemotherapeutic agents, which can reduce the serious adverse reactions caused by chemotherapy, have attracted considerable attention in clinical treatment. Nevertheless, it is important to investigate the safety challenges associated with immunotherapy and targeted therapy. According to the KEYNOTE-040 trial, treatment with pembrolizumab significantly reduced the risk of death compared with cetuximab monotherapy (hazard ratio = 0.56). The safety profile of immunotherapy combined with targeted single-drug therapy is currently being investigated, with limited published clinical data thus far. Among them, the median PFS of immunotherapy-naïve patients treated with nivolumab combined with cetuximab was 6.0 months (5). The overall safety was good, with fatigue (13%) and skin rash (4.4%) being the most common adverse reactions. The preliminary results of another phase II clinical trial (NCT03082534) showed that pembrolizumab combined with cetuximab exerts a considerable therapeutic effect on platinum-refractory patients with R/M HNSCC, with a median PFS of 8.2 months (6). Similarly, rash was the most commonly recorded immune-related adverse reaction. In the present case, the severity of dermatitis also increased during the combination therapy. As an immunoglobulin G1 (IgG1) molecule, cetuximab can induce antibody-dependent cellular cytotoxicity, in addition to blocking the activation of EGFR. Subsequently, it generates specific T cells to produce a sustained immune response. This effect is thought to be tumor immune infiltration induced by cetuximab, thereby restoring the immune suppression of the HNSCC tumor microenvironment (7, 8). The nimotuzumab and cetuximab used in this case are anti-EGFR extracellular IgG1 antibodies shown to induce the production of EGFR-specific T cells (9).

The subsequent intense immunotherapy-related response of the patient (including adverse reactions and tumor control) may be related to the activation of the immune system and promotion of the immunotherapy response by nimotuzumab. Zhou et al. retrospectively analyzed the efficacy and survival data of 4,971 patients who had received immunotherapy. They found that patients who developed adverse reactions benefited from the treatment in terms of OS and PFS (hazard ratios = 0.54 and 0.52) compared with those who did not report adverse effects (10). It is thought that adverse reactions related to immunotherapy may be linked to immune initiation triggered by tumor antigens released after treatment or humoral immune disorders (11, 12). More basic and prospective clinical research studies are warranted to further investigate the mechanism involved in this process.

### How to Improve the Anti-Tumor Activity of Immunotherapy? How Can Immunotherapy and Other Treatments Be Combined to Release More Antigens for the Activation of the Immune System?

In the present case, the combination of radiotherapy, immunotherapy, and targeted therapy eventually resulted in an excellent curative effect, thereby preventing the risk of disease hyperprogression. Could this be a new approach to immunotherapy?

Improving the immune microenvironment and activating the immune response are currently the main methods used to enhance the anti-tumor activity of immunotherapy. In this case, the addition of an EGFR monoclonal antibody induced the production of specific T cells, which could stimulate the response of the patient to immunotherapy. In a recent study, the combination of indoleamine 2,3-dioxygenase-1 (IDO1) inhibitors and PD-L1 also achieved some initial promising results in the treatment of HNSCC (13).

In addition, the combination of immunotherapy with radiotherapy or chemotherapy is also widely used. Studies have shown that PD-L1 is upregulated within 24–48 h after radiotherapy, activating the immune microenvironment (14, 15). At present, research studies on chemoradiotherapy combined with immunotherapy using different PD-1/PD-L1 monoclonal antibodies are ongoing. Among them, KEYNOTE-412 (NCT02586207), which is study combining pembrolizumab, radiotherapy, and chemotherapy, has demonstrated the safety and feasibility of this regimen. Furthermore, large-scale phase III clinical studies are also underway.

## Conclusion

In the present case, the combination of immunotherapy with radiotherapy resulted in better tumor remission. These results suggest that patients with HNSCC may benefit from a therapeutic strategy combining immunotherapy with radiotherapy or chemotherapy.

## Data Availability Statement

The datasets presented in this study can be found in online repositories. The names of the repository/repositories and accession number(s) can be found in the article/supplementary material.

## Ethics Statement

The studies involving human participants were reviewed and approved by The Fifth Affiliated Hospital of Guangzhou Medical University. The patients/participants provided their written informed consent to participate in this study. Written informed consent was obtained from the individual(s) for the publication of any potentially identifiable images or data included in this article.

## Author Contributions

Case report design: all authors. XS and HN contributed to the content of the article. Data collection and patient follow-up: HN and TC. Drafting of the manuscript: HN and TC. KH, CJL, and WG reviewed the literature and clinical data. All authors contributed to the article and approved the submitted version.

## Conflict of Interest

The authors declare that the research was conducted in the absence of any commercial or financial relationships that could be construed as a potential conflict of interest.
